# Hybrid Tau-PET/MRI study: Quantitative susceptibility mapping in progressive supranuclear palsy and its correlation with Tau-PET

**DOI:** 10.1007/s00259-025-07600-9

**Published:** 2025-11-01

**Authors:** Fiona Weih, Michael Rullmann, Dylan Henssen, Philipp M. Meyer, Thies Jochimsen, Andreas Schildan, Jost-Julian Rumpf, Matthias Brendel, Matthias L. Schroeter, Joseph Classen, Henryk Barthel, Osama Sabri, Solveig Tiepolt

**Affiliations:** 1 Department of Nuclear Medicine, University of Leipzig, Leipzig, Germany; 2 Department of Neurology, University of Leipzig, Leipzig, Germany; 3 Department of Nuclear Medicine, University Hospital of Munich, LMU Munich, Munich, Germany; 4 Munich Cluster for Systems Neurology (SyNergy), Munich, Germany; 5 German Center for Neurodegenerative Diseases (DZNE), Munich, Germany; 6 Max Planck Institute for Human Cognitive and Brain Sciences, Leipzig, Germany; 7 Clinic for Cognitive Neurology, University Hospital Leipzig, Leipzig, Germany; 8 Clinic for Nuclear Medicine, City Hospital Dessau, Dessau, Germany

**Keywords:** PET, MRI, QSM, Tau, PSP

## Abstract

**Introduction:**

The atypical Parkinsonian disorder progressive supranuclear palsy (PSP) forms a diagnostic challenge, resulting in frequent misdiagnosis and delay in treatment. Although structural MRI can detect PSP signs at more advanced stages, emerging diagnostic tools such as tau-PET and quantitative susceptibility mapping (QSM) may allow for earlier detection. This exploratory study aimed to investigate differences in QSM data of patients with PSP and healthy controls (HCs) and for the first time assess possible correlations between QSM and tau-PET data in patients with PSP to explore the relationship between tau aggregation and iron susceptibility.

**Material and methods:**

We retrospectively investigated differences in susceptibility values of brain structures, as assessed by QSM, between 11 HCs and 31 patients with PSP [Richardson’s syndrome (PSP-RS): *n = *14; other subtypes (PSP-nonRS): *n = *17]. Additionally, we examined co-registered [^18^F]PI-2620 PET and QSM data in the 31 patients with PSP to explore the relationship between tau accumulation and susceptibility changes.

**Results:**

Compared to HCs, patients with PSP showed higher QSM values in left nucleus caudate (*p* = 0.04) and bilateral dentate nucleus (*p* = 0.04, *p* = 0.01). Patients with the subtype PSP-RS showed higher QSM values than HCs in left dentate nucleus (*p* = 0.02). The association between the patients’ QSM and tau-PET data showed a significant positive correlation.

**Conclusion:**

These results suggest distinct patterns of regional iron accumulation in patients with PSP and its subtypes and support an association between iron and tau pathology. The data encourage further investigation in longitudinal studies and validation in larger cohorts to examine the value of QSM as a possible diagnostic biomarker.

**Supplementary Information:**

The online version contains supplementary material available at 10.1007/s00259-025-07600-9.

## Introduction

Progressive supranuclear palsy (PSP), one of the most common subtypes of atypical parkinsonian syndromes, is a tauopathy marked by neurostructural changes that lead to a rapid decline of cognitive and motor function, which is accompanied with a significant decrease in health-related quality of life [1]. Yet, the diagnosis of PSP remains complicated due to the absence of reliable, disease-specific biomarkers and variable clinical presentations [1, 2]. PSP is frequently misdiagnosed as idiopathic Parkinson’s disease (PD), which has an approximately 30-fold higher prevalence, or as Alzheimer’s disease and other cognitive disorders [2]. To further complicate the diagnostic challenge of PSP syndrome, its heterogeneous clinical presentation and the varying neuropathological features have led to the development of clinical sub-types, each characterized by a leading compliant. The most common clinical subtype, Richardson’s syndrome (PSP-RS), is characterized by a levodopa-unresponsive axial-predominant parkinsonism, early postural instability, vertical supranuclear gaze palsy, dysarthria, executive dysfunction and behavioural changes [3]. Other clinical subtypes include e.g. patients with predominant parkinsonism features (PSP-P), patients with pure akinesia and freezing of gait (PSP-PAGF), patients with concomitant corticobasal syndrome (PSP-CBS), patients who present with speech and language deficits (PSP-SL), and patients who present with predominant PSP symptoms and frontotemporal dysfunction (PSP-FTD), cerebellar ataxia (PSP-C) or primary lateral sclerosis (PSP-PLS) [4].

Structural MRI is an established diagnostic tool which is able to detect late stage signs, such as mesencephalon atrophy [5, 6]. However, these signs often fail to reliably distinguish PSP syndrome from its differential diagnoses at an early stage of the disease [7]. Tau-PET has emerged as a promising tool for antemortem PSP diagnosis by enabling in vivo detection of tau aggregates [8]. Given that PSP is a tauopathy, a non-invasive method to detect intracellular tau deposits in the brain, is a promising strategy. Studies suggest that tau-PET has higher sensitivity for PSP detection than conventional MRI [9]. Another alternative approach for PSP identification involves Quantitative Susceptibility Mapping (QSM), an advanced MRI technique that quantitatively assesses magnetic susceptibility, providing insight into tissue composition and disease states [10]. QSM is able to evaluate iron deposition in the brain [11], which correlates with neurodegeneration and may serve as a biomarker for various neurological conditions [12]. As iron accumulation is also observed in PSP, QSM might be an alternative biomarker next to tau PET imaging [13]. Studies have examined the ability of QSM to distinguish PSP from clinically similar conditions like PD and multiple system atrophy (MSA) by detecting distinct patterns of iron accumulation. In PSP, the red nucleus and globus pallidus show higher susceptibility than in PD, MSA, and controls, while the caudate nucleus, putamen, and substantia nigra exhibit higher susceptibility than in PD, with overall a higher iron accumulation in PSP patients [14, 15]. Despite being promising, only the putaminal rim sign has been suggested as a specific magnetic susceptibility biomarker that can be used in clinical practice to diagnose MSA at 1.5 T MRI scanning systems [16].

This exploratory study set out to combine tau-PET with QSM using a single-session, hybrid PET/MRI system. We compared QSM data of patients with PSP and healthy individuals to identify possible differences and assessed the relationship between tau aggregation and susceptibility. We hypothesised that (1) QSM would reveal susceptibility differences in deep brain nuclei distinguishing patients with PSP from healthy individuals and (2) tau pathology and iron accumulation would be correlated.

## Material and methods

### Participants

We retrospectively analysed QSM data of 31 patients with PSP (PSP-RS: *n = *14; other subtypes (PSP-nonRS): *n = *17) and 11 healthy controls (HCs). The PSP-nonRS group consists of patients with PSP-P: *n = *7, PSP-F: *n = *3, PSP-SL: *n = *3, PSP-PAGF: *n = *2, and PSP-C: *n = *2. The HCs correspond to the healthy study population described elsewhere [17]. The PSP group were scanned on the same PET/MR system and with identical acquisition protocol as the HCs. The study population demographics and clinical data are summarized in Table 1. As a clinical marker the PSP rating scale was completed by 5 patients with PSP-RS and by 7 patients with PSP-nonRS. The study was conducted in accordance with the Declaration of Helsinki, and approved by regulatory authorities and the ethics committee of LMU Munich (application numbers 17–569 and 19–022) [18]. All patients provided written informed consent.

**Table 1:** Study population demographics. PSP-RS = Patients with progressive supranuclear palsy Richardson’s syndrome; PSP-nonRS = Patients with progressive supranuclear palsy of other subtypes

	PSP-RS (*n = *14)	PSP-nonRS (*n = *17)	Healthy controls (*n = *11)	*p*
Sex [female/male]	6/8	7/10	7/4	0.46^†^
Age [years]	71 ± 8	71 ± 8	65 ± 3	0.03^§^
PSP rating scale [unitless]	43 ± 15	25 ± 15	/	0.10^‡^
Disease duration [months]	50 ± 29	53 ± 34	/	1^‡^

Values are given as mean ± standard deviation

^†^Fisher’s exact test

^§^Kruskal–Wallis test

^‡^Mann–Whitney U test

### PET data acquisition and processing

Dynamic [^18^F]PI-2620 PET data (295 ± 8 MBq) were acquired from 0 to 60 min post-injection (p.i.) using a simultaneous PET/MR scanner (Siemens mMR, Siemens Healthineers, Erlangen, Germany) with standard acquisition parameters as described elsewhere [8]. The dynamic images underwent motion correction and co-registration to their individual T1-weighed MR images. Kinetic modeling (MRTM2) was applied to generate distribution volume ratio (DVR) parametric images in PMOD (PMOD Technologies LLC, Fällanden, Switzerland) using the inferior cerebellum as reference region [18, 19].

### MR data acquisition and processing

Structural imaging was performed using a T1-weighted three-dimensional MP2RAGE sequence with 1 mm isotropic resolution (repetition time (TR) = 5000 ms; echo time (TE) = 2.98 ms; inversion time (TI) 1 = 700 ms; TI2 = 2500 ms; field of view (FOV) = 256 mm*256 mm; acquisition time = 8.5 min) [20]. To acquire QSM data the differences in local field perturbation resulting from the magnetic susceptibility distribution of the brain were measured using a 3D high-resolution spoiled gradient echo (GRE) sequence with the following parameters: 0.8 mm isotropic voxels; TR/TE = 30/20 ms, alpha = 15°, bandwidth = 220 Hz/px, FOV = 205 mm*186 mm, acquisition time = 11.5 min [17]. QSM data, consisting of a magnitude sequence and a phase sequence, were processed retrospectively and matched to the individual T1-weighted MR images in PMOD. Reference regions were manually drawn at the posterior ends of the lateral ventricles bilaterally. This region was selected because it provides a stable and commonly used internal reference with relatively low susceptibility variance and minimal structural pathology, particularly in neurodegenerative diseases [21, 22]. The mean QSM value of these cerebrospinal fluid (CSF) reference regions was subtracted from the QSM values of the brain volume of interest (VOI). QSM values (ppm) ranges (1st and 3rd quartile) of healthy volunteers are typically around [−0.018; 0.028] in cortical, [0.023; 0.129] in subcortical and [−0.027; 0.012] in cerebellar regions. Brain VOIs were generated using anatomical templates [23–25]. The regions were defined a-priori based on literature and included bilateral nucleus caudate, putamen, pallidum, red nucleus, substantia nigra, subthalamic nucleus, and dentate nucleus [14, 15, 26]. This selection of regions is not extensive in covering all sites possibly affected by PSP but aims to acknowledge common early vulnerability in PSP subtypes by covering the pallido-nigro-luysian axis [26].

### Statistics

Statistical analysis was performed using R [27]. Demographic variables were tested for normal distribution using Shapiro–Wilk tests. As most of the data was not normally distributed, we used non-parametric tests for the evaluation, i.e. Kruskal–Wallis and Mann–Whitney-U tests for group comparisons. Differences in frequency distribution were tested using Fisher’s exact test. Relationships of clinical markers were assessed using Spearman correlation analyses. To account for the hierarchical structure of the data and repeated measures across VOIs, we conducted linear mixed-effects models (LME). In the case of significant group differences in demographic parameters, we included that parameter as potential nuisance factors in the LME models. To assess group-level effects, an LME model was applied with QSM as the dependent variable and fixed effects for disease group, VOI, their interaction (disease × VOI) and potential nuisance factors. A random intercept for each subject was included to account for repeated measurements across multiple VOIs within the same individual. This approach estimates VOI-specific group differences while controlling for within-subject correlations and potential nuisance factors. We report VOI-level estimates as exploratory analyses, although the overall main effect or the group × VOI interaction might not reach statistical significance. The analyses are intended to illustrate potential regional patterns, but are not considered confirmatory. Associations between QSM and DVR (tau-PET load) were assessed using an LME model with QSM as the dependent variable, DVR and potential nuisance factors as fixed effects and a random intercept for each subject to account for repeated VOI measurements. In addition, a DVR × VOI interaction was tested to evaluate whether the slope of the DVR–QSM relationship differed across regions. Significance of fixed effects was evaluated using *F*-tests. Significance level was set at *p* < 0.05.

## Results

**Fig. 1:**
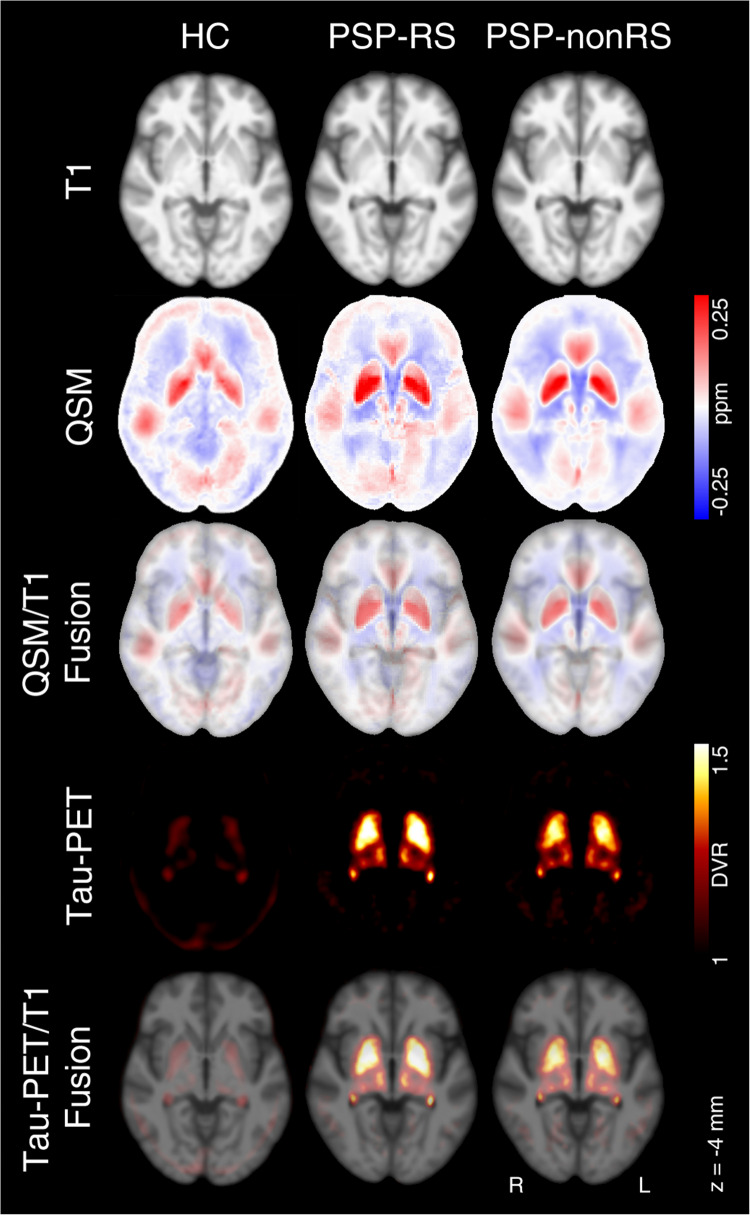
Mean images of T1-weighted MR data (top row), of quantitative susceptibility mapping (QSM, second row) and tau-PET (forth row) and as well as their superimposition (row 3 and 5) for the group of healthy controls (HC), patients with progressive supranuclear palsy with Richardson syndrome (PSP-RS) and other subtypes (PSP-nonRS). Tau-PET of HC are shown solely for reasons of visually comparison. Data is presented in MNI space (z = 4 mm). DVR – distribution volume ratio; L – left; R – right

Of 31 patients with PSP, 14 exhibited a clinical PSP-RS manifestation. The analysis compared the entire PSP group, as well as the subgroups PSP-RS and PSP-nonRS, to the healthy control group. Figure 1 displays mean images for T1, QSM and tau-PET across the PSP subgroups and controls.

**Fig. 2:**
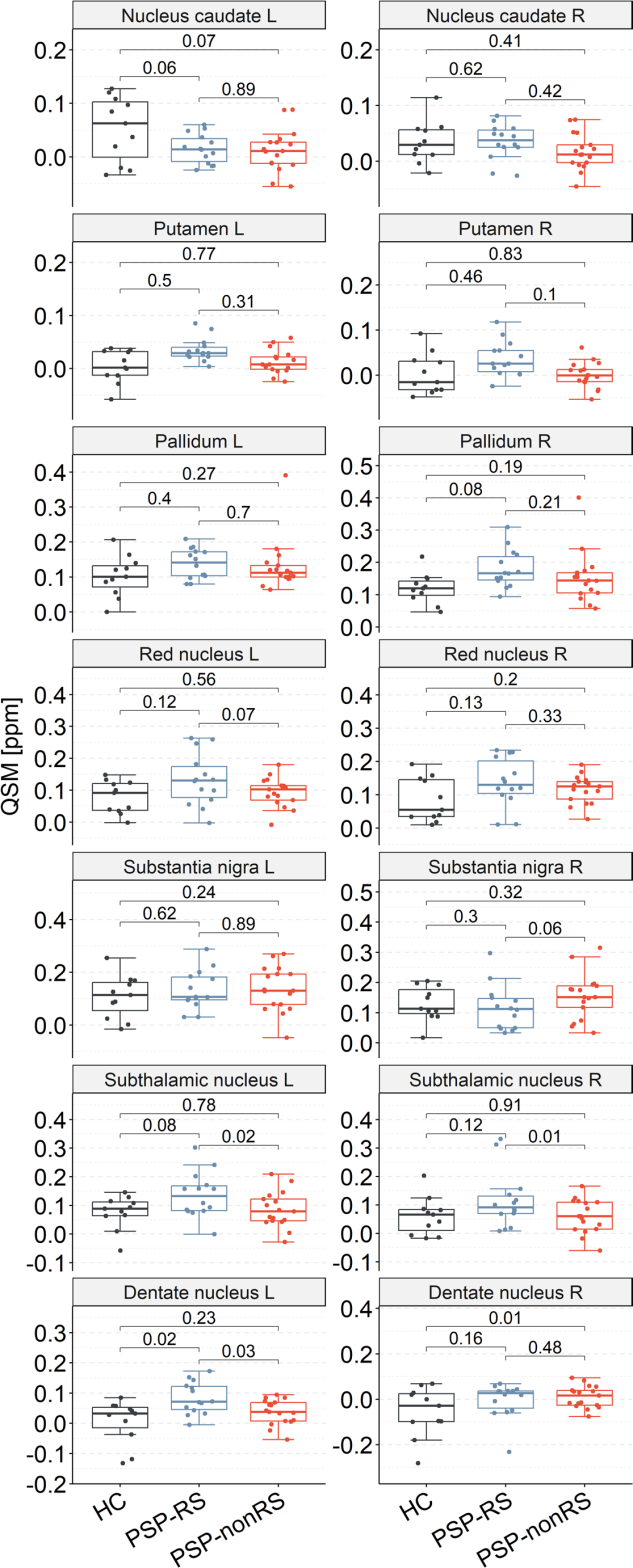
Group comparison of quantitative susceptibility mapping (QSM) values between healthy controls (HC), patients with progressive supranuclear palsy Richardson’s syndrome (PSP-RS), and patients of other subtypes (PSP-nonRS) for seven brain regions bilaterally, including caudate nucleus putamen, pallidum, red nucleus, substantia nigra, subthalamic nucleus, and dentate nucleus. FDR-corrected p values derived from the mixed-effects model including age as covariate and subject as a random intercept. L – left; R – right

To assess group differences in QSM values, we conducted an LME analysis, with age as a covariate to adjust for age differences between patients and HCs. Neither the overall main effect of group across VOIs (*p* = 0.097) nor the group × VOI interaction (*p* = 0.051) reached statistical significance. VOI-specific contrasts between HCs and patients with PSP are reported in Table 2. After FDR correction, bilateral dentate nucleus (left: estimate = −0.05 ppm, *p* = 0.04, Cohen’s d = −0.89; right: estimate = −0.05 ppm, *p* = 01, Cohen’s d = −0.78) and left nucleus caudate (estimate = 0.04 ppm, *p* = 0.04, Cohen’s d = 0.93) showed a significant group difference. All other VOIs did not survive FDR correction. When analysing only patients with PSP-RS versus HCs, we found the left dentate nucleus (*p* = 0.02) with significant higher QSM values (Fig. 2). In patients with PSP-nonRS compared to HCs, we observed significant higher QSM values in HC in the right dentate nucleus (*p* = 0.01). A direct comparison between PSP subtypes revealed significant higher QSM values in patients with PSP-RS in the bilateral subthalamic nucleus (*p* = 0.02; *p* = 0.01) and left dentate nucleus (*p* = 0.03, Fig. 2, Supplemental Table 1).

**Table 2:** Group comparison of quantitative susceptibility mapping (QSM [ppm]) values between healthy controls (HC) and all patients with progressive supranuclear palsy (PSP) showing mean ± standard deviation values, p-value, estimated differences, 95% confidence intervals and effect sizes (Cohen’s d). derived from the mixed-effects model including age as covariate and subject as a random intercept

Volumes of interest (VOI)	Healthy controls (HC)	Patients with PSP	p (FDR-corrected)	Estimate, confidence interval	Effect size
Nucleus caudate left	0.052 ± 0.061	0.013 ± 0.034	0.04*	0.045 (0.002, 0.088)	0.93
Nucleus caudate right	0.034 ± 0.037	0.025 ± 0.033	0.51	0.014 (−0.029, 0.057)	0.27
Putamen left	0.004 ± 0.031	0.023 ± 0.025	0.53	−0.014 (−0.057, 0.029)	−0.73
Putamen right	0.003 ± 0.045	0.016 ± 0.037	0.71	−0.008 (−0.051, 0.035)	−0.35
Pallidum left	0.102 ± 0.058	0.134 ± 0.061	0.23	−0.026 (−0.069, 0.017)	−0.52
Pallidum right	0.120 ± 0.047	0.165 ± 0.071	0.07	−0.040 (−0.083, 0.003)	−0.69
Red nucleus left	0.078 ± 0.051	0.111 ± 0.066	0.20	−0.028 (−0.071, 0.015)	−0.53
Red nucleus right	0.084 ± 0.065	0.126 ± 0.058	0.10	−0.036 (−0.079, 0.007)	−0.70
Substantia nigra left	0.107 ± 0.081	0.135 ± 0.079	0.30	−0.023 (−0.066, 0.020)	−0.36
Substantia nigra right	0.129 ± 0.057	0.137 ± 0.076	0.92	−0.002 (−0.045, 0.041)	−0.11
Subthalamic nucleus left	0.076 ± 0.058	0.108 ± 0.073	0.22	−0.027 (−0.070, 0.016)	−0.46
Subthalamic nucleus right	0.060 ± 0.066	0.085 ± 0.082	0.39	−0.019 (−0.062, 0.024)	−0.31
Dentate nucleus left	0.006 ± 0.072	0.058 ± 0.052	0.04*	−0.046 (−0.089, −0.003)	−0.89
Dentate nucleus right	−0.055 ± 0.109	0.004 ± 0.063	0.01*	−0.054 (−0.097, −0.011)	−0.78

^*^FDR-corrected *p*-values indicate significance after multiple comparison adjustment

**Fig. 3:**
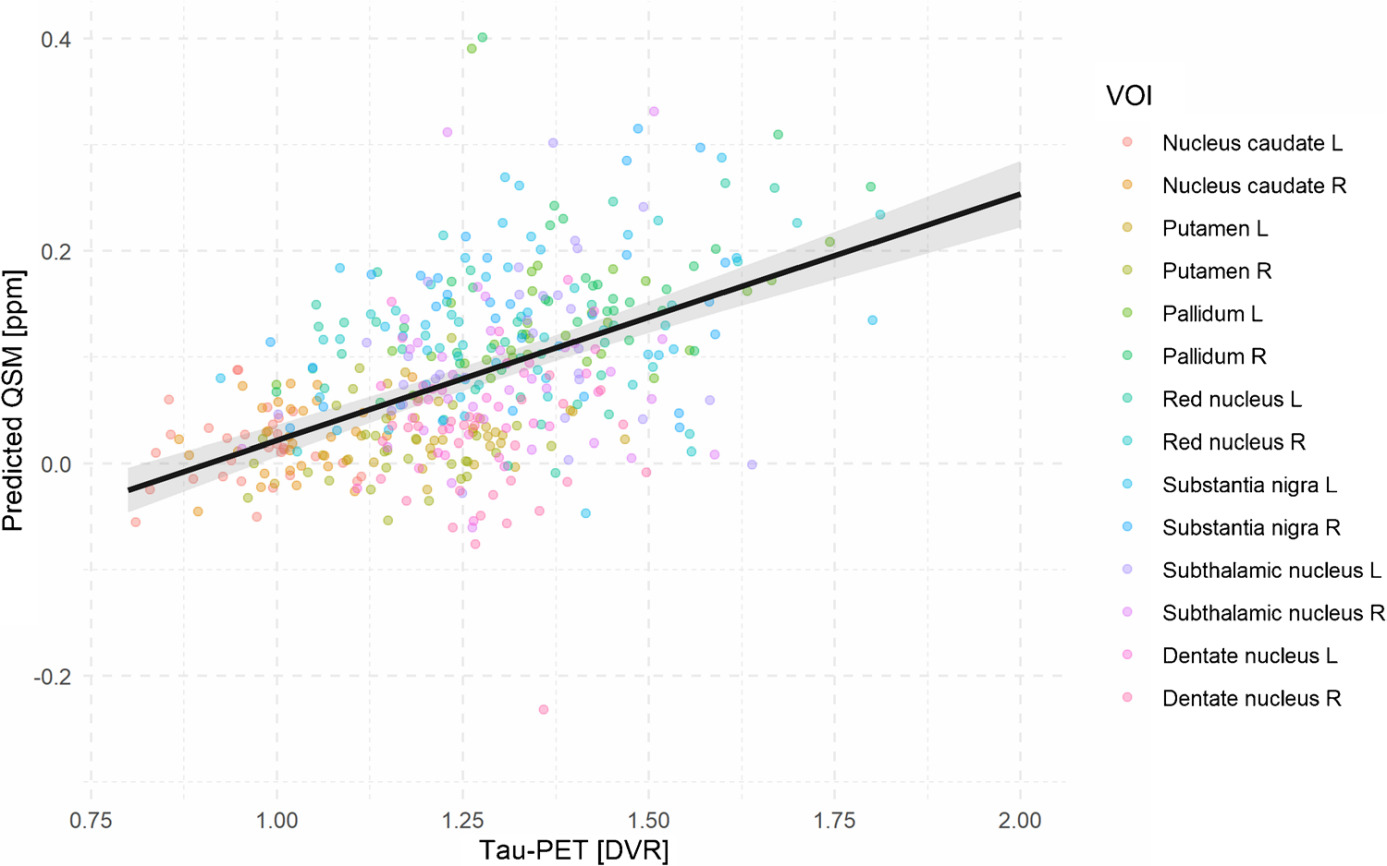
Association between quantitative susceptibility mapping (QSM) and Tau-PET values for the combined group of patients with progressive supranuclear palsy (PSP), the subgroup of patients with Richardson’s syndrome (PSP-RS), and the subgroup of patients of other subtypes (PSP-nonRS. Scatter points represent individual volume of interest (VOI) measurements (colored by region) across subjects. The black line shows the linear mixed-effects model prediction of QSM as a function of DVR, adjusted for age, with the shaded area indicating the 95% confidence interval. The model revealed a significant overall effect of DVR on QSM (*p* = 0.024), while the DVR × VOI interaction was not significant, indicating a consistent association across VOIs. L – left; R – right

To complement the regional correlation analyses, we applied LME modeling to evaluate the overall relationship between susceptibility and tau load across all VOIs and included age as a potential nuisance factor. The model revealed a significant main effect of DVR on QSM (F(1, 410) = 132.7, *p* < 2*10^–16^), indicating that higher tau load was consistently associated with higher susceptibility values (Fig. 3). However, the DVR × VOI interaction was not significant (F(13,385) = 0.52, *p* = 0.92), suggesting that this association was consistent across regions. Importantly, the DVR x PSP subtype interaction did not reach significance (F(1,402) = 0.42, *p* = 0.52), indicating a consistent association across PSP subtypes.

**Fig. 4:**
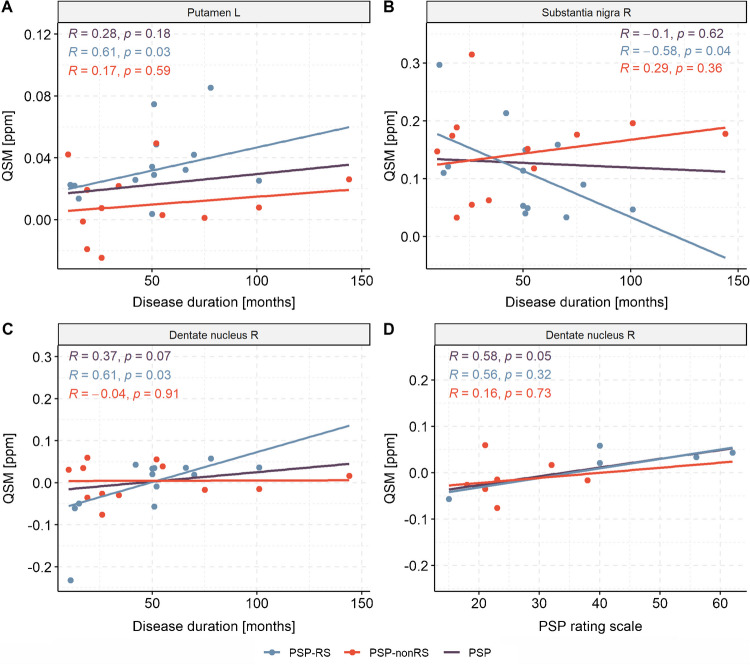
Associations between quantitative susceptibility mapping (QSM) and clinical markers such as disease duration (**A**, **B**, **C**) and PSP rating scale (**D**) for the combined group of patients with progressive supranuclear palsy (PSP), the subgroup of patients with Richardson’s syndrome (PSP-RS), and the subgroup of patients of other subtypes (PSP-nonRS). Robust regression lines are shown. R – Spearman rank correlation; L – left; R – right

When assessing the relationship of clinical markers, we identified a significant positive correlation between QSM values and disease duration exclusively in patients with PSP-RS in the left putamen (R = 0.61, *p* = 0.03) and right dentate nucleus (R = 0.61, *p* = 0.03), while the QSM values in the right substantia nigra showed a negative correlation with the disease duration (R = −0.58, *p* = 0.04). The PSP rating scale presented a significant positive correlation with QSM values in the right dentate nucleus across all patients with PSP (R = 0.58, *p* = 0.05) (Fig. 4).

## Discussion

In this study, we investigated magnetic susceptibility changes in patients with PSP compared to HCs, and examined the correlation between QSM values and tau-PET data in the PSP group.

Consistent with previous literature demonstrating increased iron levels in parkinsonian disorders, our findings indicate elevated mean magnetic susceptibility values in specific deep gray matter structures in patients with PSP and the subgroup of patients with PSP-RS compared to healthy individuals [14, 15, 28–30]. Specifically, we observed significantly increased QSM values in the left nucleus caudate and bilateral dentate nucleus in the PSP group compared to HCs. This aligns with studies using various iron-sensitive MRI techniques, including QSM, R2*, and susceptibility-weighted imaging (SWI), which consistently report increased iron concentration or related signals in the nucleus caudate and dentate nucleus of patients with PSP compared to healthy controls and other parkinsonian disorders [13, 15, 31]. Pathologically, patients with PSP are known to manifest increased iron concentrations in basal ganglia like the nucleus caudate and cerebellar dentate nucleus. Our finding of increased susceptibility in iron-sensitive MR sequence in the left nucleus caudate and bilateral dentate nucleus supports the documented increase in iron concentration, which may be related to ferritin deposits [28]. While our study identified the left nucleus caudate and bilateral dentate nucleus as regions with increased QSM in PSP relative to controls, some preceding studies have suggested the red nucleus and substantia nigra as regions with the highest susceptibility values in PSP compared to HCs [14, 29, 30]. Our results for red nucleus and substantia nigra couldn’t confirm these findings. Differences in sample size and reliability of the PSP diagnosis across studies could contribute to variations in the magnitude or statistical significance of findings across different regions.

When examining the PSP-RS subtype, we found increased QSM values in the left dentate nucleus compared to HCs, while the investigation of the PSP-nonRS subgroup showed a significant difference in QSM values in the right dentate nucleus compared to HCs. The literature specifically analysing QSM values in different subtypes of PSP is relatively limited. Increased QSM values in dentate nucleus in patients with PSP-RS compared to HCs are in alignment with some previous findings [13]. The dentate nucleus and red nucleus are interconnected via the dentatorubrothalamic tract, which has been shown to be severely degenerated in autopsy-confirmed PSP-RS [13]. The finding of increased susceptibility in the dentate nucleus in PSP-RS is thus consistent with the known pathology of this variant and could also suggest an explanation for the finding in the PSP-nonRS group. The comparison of the two subgroups showed significant differences in QSM values in bilateral subthalamic nucleus and left dentate nucleus. It might be assumed that QSM rather differentiates between patients with PSP-RS and patients with PSP-nonRS than between HCs and patients with PSP-nonRS.

Furthermore, our study explored the relationship between iron sensitive QSM MR imaging and tau pathology PET imaging readouts. To our knowledge, this is the first study analysing the correlation of QSM values and tau-PET values of multiple brain regions of patients with PSP. We found a positive correlation of individual QSM values to tau-PET data across all regions and subtypes. This finding is noteworthy as it suggests a potential co-localization or pathophysiological intersection of iron and tau pathology in PSP. The interaction of brain iron and tau is complex as both components seem to influence each other. Iron, which is known to be increased in neurodegeneration, can possibly influence tau phosphorylation and aggregation through multiple direct and indirect pathways and can induce the aggregation in hyperphosphorylated tau [12, 32]. Pérez et al. suggested that this modulation of the formation of tau aggregates by iron could be a particular characteristic in PSP [33]. The positive correlation of QSM values and the patients’ individual tau-PET data could be due to a co-localization of iron and tau pathology as already assumed by Wang et al. [34]. It can also be attributed to pathophysiological intersections of iron and tau in PSP as suggested by interactions of the two components observed in-vitro [33]. It is important to acknowledge potential off-target binding effects as confounding factor because increased tau-PET values may not solely reflect tau aggregation but could also be influenced by off-target binding effects e.g. provoked by local iron particularly in the basal ganglia [11, 12]. However, newer generation tau-tracers, including [^18^F]PI-2620, exhibit improved characteristics regarding low affinity to non-target sites compared to earlier generation tau tracers [35]. Brendel et al. assessed [^18^F]PI-2620 as a biomarker in PSP performing postmortem autoradiography in deep nuclei in samples of patients with PSP-RS and HCs and only observed minor elevation above a DVR of 1.0 in HCs, suggesting low off-target binding for [^18^F]PI-2620 in PSP target regions [8].

Our findings, both in the QSM analyses and the correlation of QSM and Tau-PET, show lateralized results. While there hasn’t been a structured exploration of potential asymmetric pathology in PSP to our knowledge, certain studies and case reports described similar asymmetry in clinical presentations as well as in cortical hemispheric morphology and tau pathology [36, 37]. Further studies are needed to explore this phenomenon.

This study represents an initial exploratory investigation into the relationship between tau PET and QSM in patients with PSP. While our findings suggest a regionally consistent association between susceptibility and tau load, they also raise the possibility that combining both modalities could offer complementary insights into disease pathology. Tau PET provides molecular specificity for aggregated tau, whereas QSM captures biophysical tissue changes, such as iron accumulation and myelin alterations, which may reflect parallel or downstream neurodegenerative processes [8, 38–40]. The integration of these modalities may therefore enhance phenotypic characterization or subtyping in PSP. However, given the exploratory nature of this work, the potential diagnostic or prognostic value of QSM and PET, either individually or in combination, remains to be determined.

Beyond its correlation with tau PET in this study, QSM offers several practical and methodological advantages. As an MRI-based method, it is non-invasive, more cost-effective and widely available compared to PET, enabling broader clinical use. Additionally, QSM detects changes in tissue susceptibility that reflect iron accumulation, demyelination and other potential microstructural alterations, thus providing complementary information to molecular imaging.

To our knowledge, studies in patients with PSP which systematically examine the correlation of QSM values and individual disease duration as well as disease severance have yet to be done. In our cohort, we found a positive correlation between QSM values in the left putamen and right dentate nucleus of the patients with PSP-RS and their disease duration, indicating a progressive iron accumulation over time. We found a negative correlation between QSM values in the substantia nigra of patients with PSP-RS and their disease duration. This effect could be attributed to a potential mechanism that early stages of PSP-RS may be characterised by increased iron deposition in the substantia nigra, which can be detected via QSM. For example, Gupta et al. found that susceptibility values in the substantia nigra were significantly higher in patients with PSP compared to those with PD, suggesting that early stages of the disease are marked by elevated iron accumulation [41]. As PSP progresses, the disease leads to substantial neuronal loss, particularly in areas such as the substantia nigra, where dopaminergic neurons are critically affected [1]. The degeneration of neurons could result in a decrease in QSM values despite the initial high levels of primarily intracellular iron deposition due to a reduced number of intact neurons, thereby explaining a negative correlation with disease duration [42]. In the entire PSP cohort, we found that QSM values in the right dentate nucleus correlate with the patients’ scores on the PSP rating scale. This effect could possibly be attributed to the dentate nucleus’s role in motor coordination which is severely affected in PSP and a prominent feature assessed in the PSP rating scale [43]. The validity of our results in the analysis of clinical parameters may be limited as we don’t have the clinical parameters of all the patients in the cohort.

Overall, our findings add to the growing body of evidence demonstrating increased iron deposition in specific deep grey matter structures in PSP, particularly in the nucleus caudate and dentate nucleus. The positive correlation between iron markers (QSM) and tau pathology (tau-PET) further highlights the complex interplay between these two key pathological features of PSP. QSM offers a quantitative method to assess brain iron accumulation, reflecting distinct topographical patterns of abnormal iron accumulation. To determine whether QSM has the potential as a biomarker for differentiating parkinsonian disorders and to understand their pathophysiology, further multimodal studies are needed, including longitudinal follow-up and validation in larger cohorts, potentially incorporating pathological confirmation, to fully elucidate the role of hybrid tau PET/QSM MR imaging in differentiating PSP variants, monitoring disease progression, and assessing treatment response.

Limitations of this study are the age difference of the two main groups, the relatively small sample size and the heterogeneous clinical phenotype of the PSP-nonRS cohort. Given that QSM values can be affected by age-related changes in brain iron content, these differences may have introduced residual confounding. We attempted to mitigate this by including age as covariates in the statistical analyses. Although, our study incorporates a larger cohort than preceding studies, it is an exploratory study with a relatively small sample size (especially regarding the PSP-RS subgroup (*n = *14)) and therefore, we still recommend larger cohorts for future studies to evaluate the significance of current findings and to possibly allow a generalization of the subgroup analyses. A further limitation is the lack of clinical data to correlate with the patients’ individual tau-PET data. As our study is a retrospective analysis, we had incoherent sets of clinical data, which only allows limited interpretation of our findings regarding the correlation of clinical data to imaging. The present analyses included multiple VOIs, which may inflate the family-wise error rate. We addressed this limitation with LME models, assessing global association across all regions and testing for group-level effects across regions while considering for within-subject correlations. These complementary models reduce the risk of inflated false positives by evaluating the effects in a unified statistical framework. Nevertheless, individual region-level findings should be interpreted cautiously and regarded as hypothesis-generating.

## Conclusion

Our study revealed significantly higher QSM values in left nucleus caudate and bilateral dentate nucleus in patients with PSP compared to HCs, as well as significantly higher QSM values in left dentate nucleus in patients with PSP-RS than HCs and right dentate nucleus in patients with PSP-nonRS than HCs. It also revealed significant differences between the subgroups of patients with PSP, PSP-RS and PSP-nonRS. To the best of our knowledge, our study is the first study investigating individual QSM and tau-PET data of patients with PSP and found a significant positive correlation over all regions and subtypes. While these findings highlight distinct regional iron changes and support an association between iron and tau pathology in the pallidum and substantia nigra, further longitudinal studies and validation in larger cohorts are warranted to examine the potential add-on of QSM alongside tau-PET imaging for diagnosis, monitoring disease progression, and assessing therapeutic efficacy.

## Supplementary Information

Below is the link to the electronic supplementary material.

Supplementary file1 (DOCX 18 KB)

## Data Availability

The datasets generated and analysed during the current study are available from the corresponding author on reasonable request. The data are not publicly available due to their containing information that could compromise the privacy of the participants.
