# Supplementary material for: Hybrid Tau-PET/MRI study: Quantitative susceptibility mapping in progressive supranuclear palsy and its correlation with Tau-PET
Source: Eur J Nucl Med Mol Imaging. 2025 Nov 1;53(3):2099–109. doi: 10.1007/s00259-025-07600-9 (PMC12860804; doi:10.1007/s00259-025-07600-9)
Supplement: Supplementary file 1 — Supplementary file1 (DOCX 18 KB) [file 259_2025_7600_MOESM1_ESM.docx]

**Hybrid Tau-PET/MRI Study: Quantitative Susceptibility Mapping in Progressive Supranuclear Palsy and Its Correlation with Tau-PET**

Fiona Weih^1*^, Michael Rullmann^1*^, Dylan Henssen^1^, Philipp M. Meyer^1^, Thies Jochimsen^1^, Andreas Schildan^1^, Jost-Julian Rumpf^2^, Matthias Brendel^3,4, 5^, Matthias L. Schroeter^6, 7^ Joseph Classen^2^, Henryk Barthel^1,8^, Osama Sabri^1^, Solveig Tiepolt^1^

^1^ Department of Nuclear Medicine, University of Leipzig, Leipzig, Germany.

^2^ Department of Neurology, University of Leipzig, Leipzig, Germany.

^3^ Department of Nuclear Medicine, University Hospital of Munich, LMU Munich, Munich, Germany.

^4^ Munich Cluster for Systems Neurology (SyNergy), Munich, Germany

^5^ German Center for Neurodegenerative Diseases (DZNE), Munich, Germany

^6^ Max Planck Institute for Human Cognitive and Brain Sciences, Leipzig, Germany

^7^ Clinic for Cognitive Neurology, University Hospital Leipzig, Leipzig, Germany.

^8^ Clinic for Nuclear Medicine, City Hospital Dessau, Dessau, Germany.

* These authors contributed equally to this work.

Corresponding author:

Solveig Tiepolt, Email address: Solveig.tiepolt@medizin.uni-leipzig.de

**Supplemental Material**

Supplemental Table 1. Group comparison of quantitative susceptibility mapping (QSM [ppm]) values between healthy controls (HC) and all patients with progressive supranuclear palsy (PSP) including their subtypes showing p-value and effect size, derived from the mixed-effects model including age as covariate and subject as a random intercept.

| **Volume of interest (VOI)** | **HC vs. PSP-RS** | **HC vs. PSP-nonRS** | **PSP-RS vs. PSP-nonRS** |
| --- | --- | --- | --- |
| nucleus caudate left | 0.06  0.83 | 0.07  0.84 | 0.89  0.09 |
| nucleus caudate right | 0.62  -0.02 | 0.41  0.49 | 0.42  0.54 |
| putamen left | 0.50  -1.19 | 0.77  -0.36 | 0.31  0.98 |
| putamen right | 0.46  -0.81 | 0.83  0.07 | 0.10  1.09 |
| pallidum left | 0.40  -0.72 | 0.27  -0.41 | 0.70  0.13 |
| pallidum right | 0.08  -1.11 | 0.19  -0.48 | 0.21  0.38 |
| red nucleus left | 0.12  -0.78 | 0.56  -0.33 | 0.07  0.61 |
| red nucleus right | 0.13  -0.77 | 0.20  -0.63 | 0.33  0.37 |
| substantia nigra left | 0.62  -0.35 | 0.24  -0.36 | 0.89  -0.04 |
| substantia nigra right | 0.30  0.21 | 0.32  -0.37 | 0.06  -0.53 |
| subthalamic nucleus left | 0.08  -0.86 | 0.78  -0.14 | 0.02*  0.74 |
| subthalamic nucleus right | 0.12  -0.63 | 0.91  0 | 0.01*  0.70 |
| dentate nucleus left | 0.02*  -1.25 | 0.23  -0.55 | 0.03*  1.02 |
| dentate nucleus right | 0.16  -0.55 | 0.01*  -0.87 | 0.48  -0.24 |

*FDR-corrected p-values indicate significance after multiple comparison adjustment.
